# A Novel Homozygous *BMP15* Mutation Causes Ovarian Dysgenesis and Primary Amenorrhea

**DOI:** 10.1210/jendso/bvae221

**Published:** 2024-12-05

**Authors:** Amitay Cohen, Raffaella Rossetti, Natan Florsheim, Abraham O Samson, Paul Renbaum, Erika Carbone, Luca Persani, Ephrat Levy-Lahad, Abdulsalam Abu-Libdeh, David Zangen

**Affiliations:** Division of Pediatric Endocrinology, Hadassah Medical Center, Jerusalem 91240, Israel; Faculty of Medicine, Hebrew University of Jerusalem, Jerusalem 9112102, Israel; Department of Endocrine and Metabolic Diseases, IRCCS Instituto Auxologico Italiano, Milan 20149, Italy; Faculty of Medicine, Hebrew University of Jerusalem, Jerusalem 9112102, Israel; Institute of Medical Genetics, Shaare Zedek Medical Center, Jerusalem 91031, Israel; Faculty of Medicine, Bar Ilan University, Safed 1589, Israel; Institute of Medical Genetics, Shaare Zedek Medical Center, Jerusalem 91031, Israel; Department of Endocrine and Metabolic Diseases, IRCCS Instituto Auxologico Italiano, Milan 20149, Italy; Department of Endocrine and Metabolic Diseases, IRCCS Instituto Auxologico Italiano, Milan 20149, Italy; Department of Medical Biotechnology and Translational Medicine, University of Milan, Milan 20133, Italy; Faculty of Medicine, Hebrew University of Jerusalem, Jerusalem 9112102, Israel; Institute of Medical Genetics, Shaare Zedek Medical Center, Jerusalem 91031, Israel; Division of Pediatric Endocrinology, Hadassah Medical Center, Jerusalem 91240, Israel; Faculty of Medicine, Hebrew University of Jerusalem, Jerusalem 9112102, Israel; Department of Pediatrics, Makassed Islamic Charitable Hospital, Jerusalem 19482, Israel; Division of Pediatric Endocrinology, Hadassah Medical Center, Jerusalem 91240, Israel; Faculty of Medicine, Hebrew University of Jerusalem, Jerusalem 9112102, Israel

**Keywords:** BMP15, primary amenorrhea, ovarian dysgenesis, infertility, primary ovarian insufficiency

## Abstract

**Context:**

Despite a growing number of studies, the genetic etiology in many cases of ovarian dysgenesis is incompletely understood.

**Objectives:**

This work aimed to study the genetic etiology causing absence of spontaneous pubertal development, hypergonadotropic hypogonadism, and primary amenorrhea in 2 sisters.

**Methods:**

Whole-exome sequencing was performed on DNA extracted from peripheral lymphocytes of 2 Palestinian sisters born to consanguineous parents. Following a *BMP15* variant identification, confirming genetic segregation studies were performed in family members. Three-dimensional (3D) modeling for BMP15 dimer and BMP15-GDF-9 heterodimer were followed by functional studies in human ovarian COV434 granulosa cells cotransfected with plasmid harboring either the variant or a wild-type (WT) control, and a second plasmid harboring a luciferase-reporter-gene with a BMP-responsive element.

**Results:**

A novel homozygous c.G959A/p.C320Y *BMP15* mutation was identified in both sisters, and segregated with the disease in the family. By 3D-structure modeling, the mutations were predicted to damage a cysteine-knot motif, disrupt BMP15 dimerization, and severely impair activation of the BMP pathway. The homologous mutation C53Y occurring and identified spontaneously in sheep results in sterility in homozygotes, mimicking the human phenotype here. A 3.8-fold decrease in BMP15 signaling was observed in vitro in cells expressing the homozygous BMP15 mutant when compared to the WT control.

**Conclusion:**

The novel homozygous missense C320Y mutation is the first homozygous human *BMP15* variant causing impaired signaling ability, which correlates with the predicted 3D-structural changes leading to ovarian dysgenesis. The homologous mutation in sheep mimics the human phenotype by infertility. Beyond genetic counseling, and considering ovarian preservation, the ovine model enables further elucidation and interventions in the BMP signaling.

Primary ovarian insufficiency (POI) affects about 3.7% of women worldwide [1]. Mild cases manifest as secondary amenorrhea for more than 4 months in women younger than 40 years, while severe cases manifest as ovarian dysgenesis (OD), primary amenorrhea, and absence of spontaneous pubertal development [2]. Genetic forms of OD stem in many cases from a disruption of the follicle pool. One of the genetic factors associated with POI is bone morphogenic protein 15 (BMP15) mutations. BMP15 is an X-linked, oocyte-specific secreted protein of the transforming growth factor β superfamily. The functional mature protein is a product of a cleaved preprotein (1-392) that consists of a signal peptide (1-17), a proregion (18-267), and a mature peptide (268-392), that contains 9 cysteines, of which 6 form a highly conserved cystine-knot motif that is characteristic of the BMP family [3].

BMP15 is expressed in the oocyte from the early secondary follicular stage up to the preovulatory stage and in mature second metaphase oocytes [4-6]. BMP15 along with its closest homologue GDF9 act in tandem and induce granulosa cell (GC) proliferation and expression of genes required for cumulus cell expansion [7, 8]. In later follicular stages, BMP15 and GDF9 are implicated in the modulation of energy metabolism and cholesterol biosynthesis in GCs, nourishing the oocyte with essential pyruvate and fatty acid metabolites via gap junctions [9]. Thus, BMP15 and GDF9 are pivotal for early folliculogenesis.

Several heterozygous missense *BMP15* mutations have been associated with primary amenorrhea, and most are located in the proprotein region. Reports of homozygous deletions in *BMP15* associated with primary or secondary amenorrhea do exist but are rare. To the best of our knowledge, no homozygous *BMP15* missense mutations in the mature region causing primary amenorrhea have been described in humans before, nor biochemically characterized. In this study, we identified and for the first time functionally characterized a novel homozygous variant of BMP15 in 2 sisters who presented with POI.

## Materials and Methods

**Figure 1.**
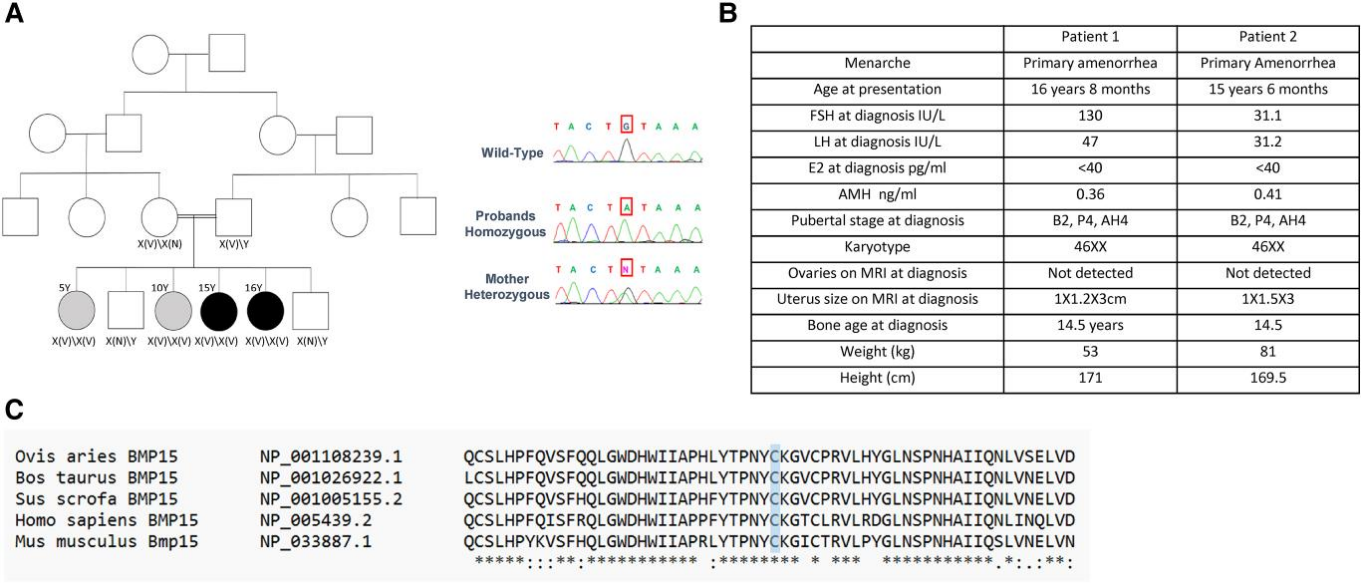
*BMP15* in a family with ovarian dysgenesis. A, Segregation of *BMP15* variants in the studied family. Solid black symbols indicate affected family members and solid gray symbols indicate prepubertal family members likely to be affected. Segregation is consistent with X-linked recessive inheritance. Genotype is indicated underneath tested family members, X(V) denotes the variant and X(N) the normal gene. To the right is the *BMP15* Sanger sequencing result. Normal control wild-type (WT) shows reference sequence. The probands are homozygous for a missense mutation in locus NM_005448:exon2:c.G959A in the *BMP15* gene on chromosome X. The mother is a carrier and the father is hemizygous for the mutation. B, Clinical characteristics of probands with primary ovarian insufficiency (POI). C, Clustal alignment analyses of *BMP15* sequence over different species commonly used as an animal model for POI. C320 residue is highlighted in light blue.

The probands are 2 sisters from a consanguineous Palestinian family referred to Hadassah tertiary center for further evaluation due to primary amenorrhea. Both sisters had uneventful medical history and presented with almost absence of spontaneous puberty at age 16.6 and 15.5 years, respectively (Fig. 1A). Physical examinations were unremarkable. Breast development was at Tanner stage II and laboratory results were consistent with hypergonadotropic hypogonadism (luteinizing hormone [47 and 31.2 IU/L, normal range [NR], 1.5-5.6 IU/L], follicle-stimulating hormone [130 and 31.1 IU/L, respectively; NR, 3.6-7.9 IU/L], and undetected estradiol [both < 40 pmol/L]). Serum antimüllerian hormone [AMH] levels 2 years after initial presentation was low in both probands (0.36 ng/mL and 0.41 ng/mL, respectively; NR 0.5-6.2 ng/mL). Karyotype was 46,XX. Bone age was delayed (14.5 years) and magnetic resonance imaging demonstrated an infantile uterus and no visible gonads in both patients. Parents had normal fertility, with no known history of delayed puberty or infertility in the extended family. Two prepubertal younger sisters were aged 5 and 10 years at the time of evaluation; their AMH levels were 0.38 ng/mL (0.26-5.4 ng/mL) and 0.08 ng/mL (2.84-7.95 mg/mL), respectively [10]. Patient clinical and imaging characteristics are shown in Fig. 1B.

### Study Approval

This study was approved by the institutional review board of the Shaare Zedek Medical Center and the National Ethics (Helsinki) Committee. Informed consent was obtained from all participants and their legal guardians.

### Genomic Analysis

DNA was extracted from peripheral blood mononuclear cells using the Flexigene DNA blood extraction kit (Qiagen GmbH). Whole-exome sequencing (WES) was performed on genomic DNA samples by Beijing Genomics Institute (BGI), using the Agilent V6 Exome capture kit and sequenced to a mean coverage of 150× (10 Gb). Bioinformatics were performed on an in-house pipeline as previously described [11]. Candidate variants were validated by Sanger sequencing using BigDye Terminator v1.1 sequencing kit and analyzed on a 3130xl Genetic Analyzer (Applied Biosystems) with software version 5.3.1.

### Structural Modeling

To model the 3-dimensional (3D) structure of the BMP15/GDF9 heterodimer (ie, cumulin) and its complex with the ectodomains of the BMPR, we used the AlphaFold Protein Structure Database for predictions, and the PyMOL Molecular Graphics System, version 2.0 (Schrödinger LLC) for superimposition and analysis. First, the predicted structure of BMP15, corresponding to the entire precursor protein (1-392), was downloaded from Alphafold (Alphafold entry O95972). The BMP15 structure was edited, and the signal peptide (1-17) and proregion (18-267) were removed. The remaining BMP15 structure corresponded to the mature peptide (268-392). Similarly, the predicted structure of the GDF9 mature peptide (349-454) was downloaded (Alphafold entry A0A7K8N8A8). Then, to model cumulin, which is the BMP15/GDF9 heterodimer, the mature peptide structures of BMP15 and of GDF9 were superimposed onto those of the homologous BMP2 homodimer (PDB ID 1ES7). This structure (PDB ID 1ES7) already contained the 2 ectodomains of BMPRI (PDB ID 1ES7) and served as a scaffold for the cumulin/BMPRI relationship. Finally, 2 ectodomains of BMPRII were imported from the activin A/activin receptor II complex (PDB ID 1NYU). This structure already contained the 2 ectodomains of BMPRII and served as a scaffold for the cumulin/BMPRII relationship.

### Construction of *BMP15* Expression Plasmids

The expression plasmids for luciferase assay were obtained from amplification of full-length human BMP15 complementary DNA sequence from the corresponding pCDNA4 vector containing either the human BMP15-MycHis wild-type (WT) or the c.959G > A (p.C320Y) mutation [32] with specific primers introducing restriction sequences for ClaI and XbaI, at 5′ and 3′ ends:

ClaI forward: 5′ CTATCGATAAGATGGTCCTCCTCAGTATTC 3′

XbaI reverse: 5′ CTTCTAGATCATCTGCAGGTACAAGACTCAGC 3′

WT and the c.959G > A variant sequences were then subcloned into the pCS2++ empty vector.

### Cell Culture and Transfections

Cells from an immortalized human ovarian GC line COV434 that maintains the characteristics of a primary granulosa cell [12] were cultured in Dulbecco's modified Eagle medium with Glutamax (DMEM-Glutamax, Thermo Fisher Scientific), supplemented with 1% penicillin-streptomycin and 10% fetal bovine serum (all from Sigma Aldrich), the maintenance medium, at 37 °C and 5% CO_2_.

The luciferase assay was performed using a BMP responsive element (BRE)-luciferase reporter, as previously described [13]. For the luciferase reporter assay, COV434 cells were seeded at a density of 7 × 10^5^ cells/well in 12-well plates in the maintenance medium. Twenty-four hours later, COV434 cells were transiently cotransfected with either WT or mutant (c.959G > A) pCS2-BMP15 expression vector (400 ng/well) and the BRE-luciferase reporter (100 ng/well) using Fugene HD (Promega), following manufacturer's instructions. A total of 10 ng per well of pRL-TK plasmid containing the Renilla luciferase gene (Promega) were cotransfected as an internal control of transfection efficiency. Each condition was transfected in triplicate. The pCS2++ empty vector was used as negative control. Twenty-four hours post transfection, the transfection medium was replaced with DMEM-Glutamax supplemented with 1% fetal bovine serum. The condition of pCS2++ empty vector treated with 10 ng/mL rhBMP4 (R&D Systems) 24 hours post transfection was used as positive control of BMP pathway activation.

### Luciferase Reporter Assay

Sixteen hours after medium replacement, COV434 cells were rinsed with ice-cold phosphate-buffered saline and then lysed with 100 μL of Passive Lysis Buffer 1× (Promega). Cell lysates from each well were centrifuged at 12 000 rpm for 2 minutes to pellet the cell debris and 20 μL of the supernatants were then assayed for luciferase activity using the Dual Luciferase Reporter Assay kit (Promega). Luminescence in relative light units (RLU) was measured for 10 seconds in a Fluoroskan Ascent instrument (Labsystems). The Firefly signals were normalized for those of Renilla for each transfection condition and the means of triplicates obtained. Results were then calculated as fold induction relative to the pCS2-BMP15 WT expression vector.

### Statistics

Luciferase reporter assays were performed 8 times in triplicate wells. All values represent the mean of fold changes in logarithmic scale vs WT ± SEM. Repeated multiple one-way analysis of variance (ANOVA) applying the Geisser-Greenhouse correction was carried out, and Bonferroni posttest was used to correct multiple comparisons (GraphPad Prism software v.8.4.2). A *P* value of less than .05 was considered statistically significant.

## Results

### Molecular Genetics

Analysis of coding and splice region variants in WES in both sisters using a model consistent with X-linked recessive or autosomal recessive inheritance revealed a novel homozygous missense variant in both affected sisters in the *BMP15* gene, c.G959A: p.C320Y (GenBank accession No. NM_005448.2, position [hg19] ChrX: 50659386, rs782609889). The variant that has not been reported in gnomAD cosegregated with the clinical phenotype of primary amenorrhea and was confirmed using Sanger sequencing. No other variants in POI-related genes have been observed. This cysteine residue is highly conserved across multiple species, as evident by performing a Clustal alignment analyses of BMP15 sequence over different species commonly used as an animal model for POI [14] (Fig. 1C). The healthy fertile father was hemizygous and the mother heterozygous. Two younger prepubertal sisters were also found to be homozygous (see Fig. 1A).

**Figure 2.**
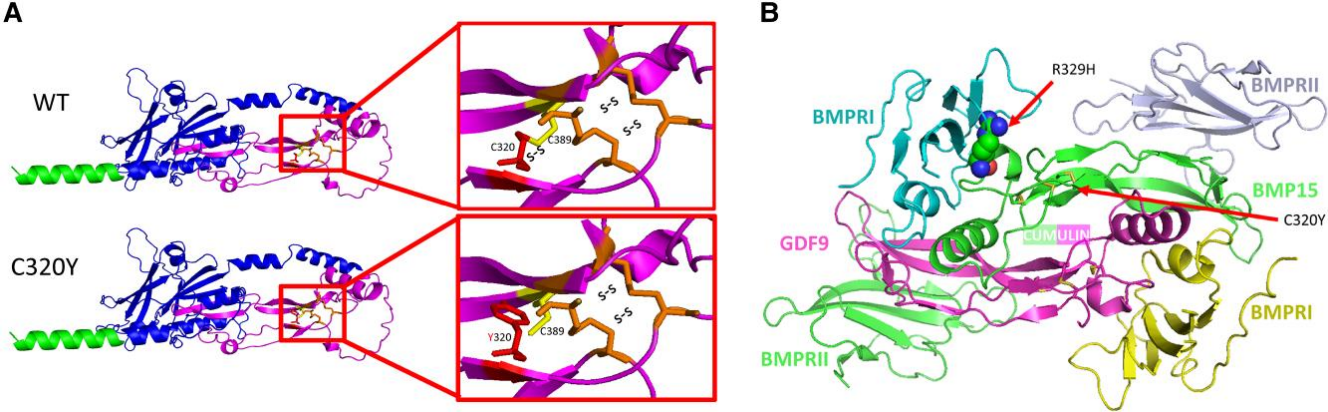
A, Predicted structural effect of C320Y variant in *BMP15*. G959A mutation substitutes a highly conserved cysteine residues in position 320 with tyrosine; left—full BMP15 protein structure includes signal peptide (green), propeptide (blue), and mature BMP15 peptide (magenta). Right—enlargement of the cysteine-rich region. Wild-type (WT) marks the normal protein sequence and structure; C320Y marks the predicted change in the mutant BMP15. Orange marks the cysteine residues and bonds within the cysteine motif, yellow marks C389 that bind WT C320 and form a cysteine bond. Red marks the C320Y substitute in the mutant *BMP15*. This structural change within a cysteine motif is disrupting a highly conserved cysteine bond and predicted to impair the protein structure and folding. B, Shown in the center are the ribbon diagrams of cumulin, a heterodimer consisting of one bone morphogenetic protein-15 (BMP15) subunit, and one growth differentiation factor-9 (GDF9) subunit, bound to the ectodomains of its receptor, the bone morphogenetic protein receptor (BMPR), a tetramer consisting of 2 BMPRI subunits, and 2 BMPRII subunits. The model is based on the structures of the BMP2/BMPR1 complex (PDB ID 1ES7) which serve as a scaffold for that of cumulin/BMPRI, and the structure of the activin A/activin receptor II complex (PDB ID 1NYU), which serves as a scaffold for that of cumulin/BMPRII. The C320Y mutation, indicated by a red arrow, disrupts the cysteine knot-motif (sticks representation), deforms the heterodimer interface of BMP15, and prevents cumulin formation. The R329H mutation, indicated by a second red arrow, exerts no effect on cumulin formation, but instead disrupts the interaction of cumulin with BMPRI, and prevents receptor activation. The model and figure were prepared using Pymol.

In tridimensional structural modeling analysis using AlphaFold Protein Structure Database, this mutation, changing the amino acid from cysteine to tyrosine in position 320 breaks the disulfide bond between C320 and C389, disrupts the highly conserved cysteine-knot motif of the mature peptide of BMP15, and induces a deformation of adjacent residues lining the cumulin interface. In turn, the deformation impedes dimerization of the BMP15 mature peptide with itself (BMP15/BMP15 homodimer), or with GDF9 (BMP15/GDF9 heterodimer-cumulin), and blocks cumulin formation (Fig. 2A). As such, the C320Y mutation disrupts the cysteine-knot motif and destabilizes the dimer-prone conformation of the BMP15 mature peptide structure (Fig. 2B). This variant was therefore classified as likely pathogenic (PM1, PM2, PP1, PP2) according to the American College of Medical Genetics and Genomics standards and guidelines [15].

### Expression and Functional Studies

**Figure 3.**
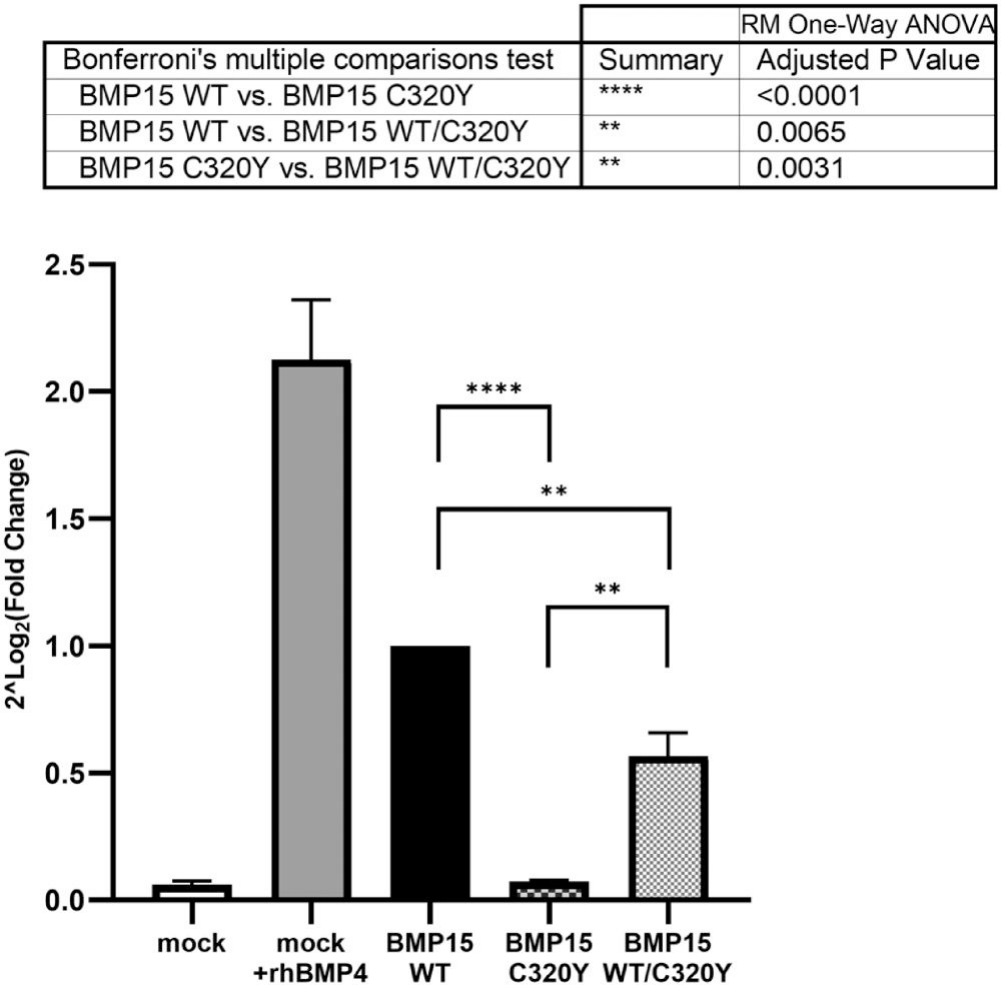
The c.959G > A (p.C320Y) variant of *BMP15* impairs the activation of the BMP pathway. The transcriptional activities of *BMP15* WT and of its variant 320Y were studied through a luciferase assay using a BMP-responsive element (BRE)-luciferase reporter in COV434 granulosa cells. Recombinant human BMP4 (rhBMP4) was used as an exogenous stimulus of the BMP pathway. Results are shown as mean of fold changes vs wild-type (WT) ± SEM of 8 independent experiments, each performed in triplicate. **P less than .001; ****P less than .0001. RLU, relative light units.

The functional consequence of the BMP15 C320Y variant on the activation of the BMP pathway was evaluated in a COV434 line of immortalized human GCs through a dual-luciferase assay that measured BMP signaling as previously reported [7]. Statistical analysis was performed by applying one-way ANOVA for repeated measures, and the resulting overall ANOVA *P* value was less than .0001 (Geisser-Greenhouse's epsilon = 0.3045; *R*^2^ = 0.8855). The statistical difference between the same transfection condition of each independent replicate was not statistically different (*P* = .6251). The transactivation competence of BMP15 on transfection of the variant was dramatically suppressed when compared to transfection with the human WT *BMP15* (WT vs C320Y; *P* < .0001) (Fig. 3). When this variant was cotransfected with an equal amount of the normal BMP15 WT construct, with the aim of mimicking the heterozygous condition, the transactivation activity was partially rescued, but the impairment remained statistically significant (WT vs WT/C320Y; *P* = .0065) when compared to the WT. On the other hand, the impairment in the heterozygous state was significantly less prominent than the severe biologic compromise in transactivation observed in the homozygous state (C320Y vs WT/C320Y; *P* = .0031) (see Fig. 3).

## Discussion

In this study we report a novel and first homozygous missense C320Y mutation in *BMP15* causing ovarian dysgenesis, and provide some molecular and functional evidence supporting the role of BMP15 in human folliculogenesis.

BMP15 is an X-linked, oocyte-specific protein secreted by the oocyte in a paracrine fashion, beginning in the early-mid secondary follicle [5]. It induces GC proliferation in human [16] and mice [17] cell lines in vitro. It does so as a homodimer by binding complexes of membrane type 1 (ALK6 or BMPRI) and type 2 (BMPR2) receptors, thereby activating mainly the Smad1/5 but also the Smad2/3 pathways. BMP15 also binds GDF9, (another highly homologous member of the TGFβ superfamily), and as a heterodimer named *Cumulin* [18], potently induces the Smad2/3 pathway in porcine and murine cell cultures, and stimulates murine cumulus expansion [19]. The novel C320Y mutation induces a deformation of both the highly conserved cysteine-knot motif of the mature peptide of BMP15 between p.C320 and p.C389 (see Fig. 2A and 2B) and of the residues lining the cumulin heterodimer interface (see Fig. 2B). The tridimensional modeling of the reported mutation indicates that while the C320Y mutation exerts only minor effects on the global BMP15 structure, it exerts a profound effect on the local structure of the BMP15 mature peptide. The local deformation prevents dimerization of the BMP15 mature peptide (268-392) with itself as a homodimer (BMP15/BMP15), or with GDF9 as the cumulin heterodimer (BMP15/GDF9). The consequent disrupted cumulin formation and action impairs both GC proliferation and folliculogenesis and is predicted to lead to the observed clinical phenotype.

The vast majority of reported *BMP15* mutations that are associated with primary or secondary amenorrhea are heterozygous and located in the proprotein region. Most studies have not simulated a truly heterozygous state, as culture cells were not simultaneously exposed to WT and mutant BMP15 [20]. In some (p.L148P, p.R138H, and p.R68W) coexpression of WT BMP15 in the same cell cultures resulted in partial rescue of luciferase action [13], which did not mimic the human clinical associated phenotype. We have recently identified a reported [13] heterozygous L148P mutation in *BMP15* in 2 sisters with primary amenorrhea. Surprisingly, the fertile mother was also found to carry a similar mutation, which puts the pathogenicity of the L148P mutation in question.

The spectrum of the clinical phenotype in *BMP15* variants between primary to only relatively late secondary amenorrhea was always challenging. The novel C320Y mutation in this study, which was homozygous, uniquely within the mature domain of BMP15, and presenting in 2 patients with an identical severe clinical phenotype (primary amenorrhea), intrigued us as a cleaner model to assess and functionally understand the role of BMP15 as a cause of OD.

**Figure 4.**
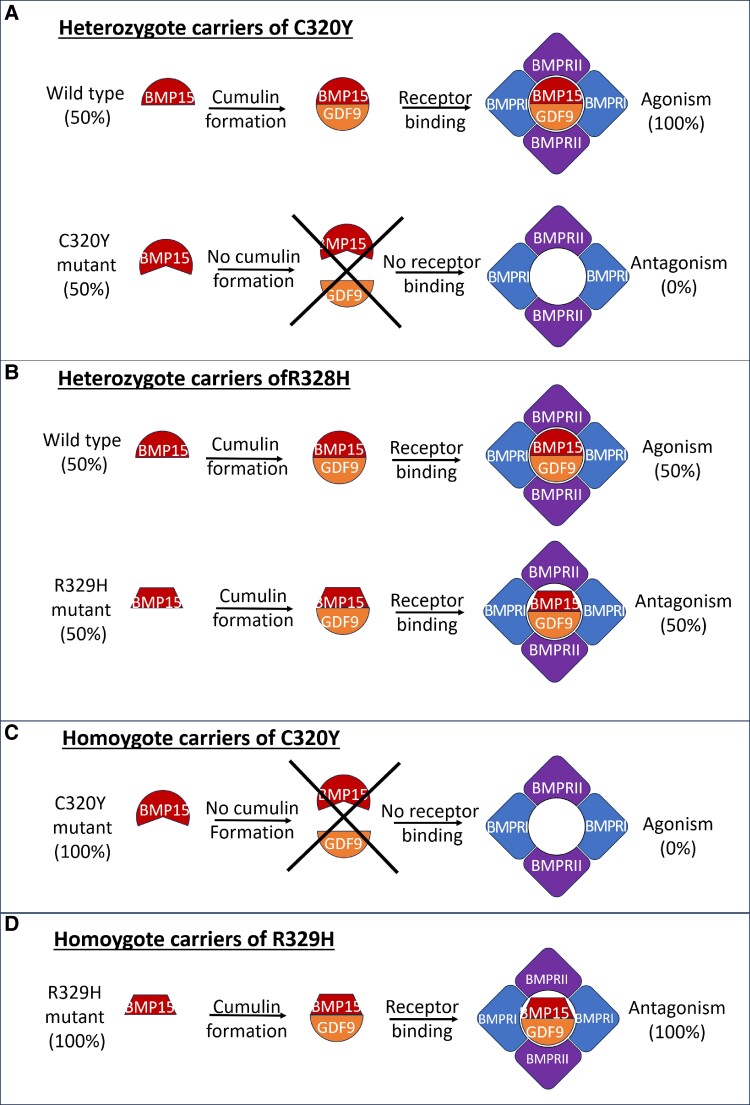
Heterozygous and homozygous carriers of *BMP15* mutations. Shown are the different effect of mutation: A, In C320Y heterozygosity, the *BMP15* gene is expressed as wild-type (WT) (50%) and mutant (50%). Only the WT dimerizes with GDF9 to produce cumulin, which can bind to the receptor, and exerts all activity (100%). The mutant cannot dimerize, cannot bind to the receptor, and exerts no effect on the receptor (0%). B, In R329H heterozygosity, the *BMP15* gene is expressed as WT (50%) and mutant (50%). Both the WT and the mutant can dimerize into cumulin, and bind to the receptor. WT cumulin (50%) is a receptor agonist, whereas mutant cumulin (50%) is an antagonist, and their combination reduces receptor activity. C, In C320Y homozygosity, the *BMP15* gene is expressed as mutant only (100%). The mutant cannot dimerize, cannot bind to the receptor, and exerts no effect on the receptor (0%). D, In R329H homozygosity, the BMP15 gene is expressed as mutant only (100%). The mutant dimerizes into a cumulin antagonist and can bind to the receptor. However, the antagonist does not activate the receptor (0%).

The clinical variability in heterozygotes is probably derived from the complex nature of BMP15 action involving homodimerization and heterodimerization, and receptor binding. The R329C and R329H BMP15 heterozygote mutants hamper Smad2/3 and Smad1/5 signaling in vitro in GCs [20-22] as all BMP15 is predicted to dimerize, to form cumulin, which binds to the receptor. However, only one-half elicits a WT cumulin response, while the other half, the mutant cumulin, presumably acts as an antagonist (Fig. 4), leading to abnormal oogenesis and ovarian development (secondary amenorrhea). In contrast, in the present reported C320Y heterozygotes (the healthy fertile mother of the 2 sisters), the mutant BMP presumably cannot dimerize into cumulin, and therefore there is no dominant negative effect. The existing WT protein does dimerize and bind the receptor with no compromise in folliculogenesis indicating that haploinsufficiency of BMP15 alone is not sufficient explanation for POI in girls with Turner syndrome [23], as the heterozygous mother in the present study had normal puberty and fertility.

In C320Y homozygotes, however, BMP15 is predicted to lose dimerization ability into cumulin and therefore cannot activate the receptor, thus producing severe primary amenorrhea and OD. To further establish this concept, we studied the BMP15 signaling in immortalized human GCs through a dual-luciferase assay and showed that indeed this change (C320Y) causes a dramatic reduction in BMP15 signaling in vitro. To the best of our knowledge, this is the first homozygous pathogenic *BMP15* mutation that was shown to decrease the signaling of BMP15 and cause primary amenorrhea in human.

Previous reports of homozygous or compound heterozygous BMP15 mutations were identified in patients with primary or secondary amenorrhea, but no functional studies addressing the effect on SMAD signaling have been performed to prove causality [24-27]. In 2 sisters with secondary amenorrhea, a compound heterozygous *BMP15* mutation R264Q/P359L was identified and was shown to affect GC proliferation in vitro [28]. In another patient with secondary amenorrhea, a homozygous frameshift in the proregion of BMP15 resulted in a truncated protein product in vitro [29]. In other reports the mutation was predicted to exert a “knockout-like” effect as proprotein region mutations causing early protein truncation were clinically associated with primary or early secondary amenorrhea [25].

Nevertheless, studying BMP15 biology was hampered by the paucity of an adequate animal model. Murine *BMP15* knockout mutations, for example, were shown to have only a minor effect on female fertility, as *bmp15*-null female mice are only subfertile, with minimal ovarian histologic defects [30]. In contrast, in sheep different BMP15 mutations were associated with sterility. These profound differences are hypothesized to stem from the mono-ovulatory nature of sheep vs the poly-ovulatory nature of mice, as BMP15 may be dispensable in the latter [31]. Remarkably, a spontaneous occurring C53Y mutation in sheep, homologous to the C320Y human mutation reported here, is responsible for the prolific phenotype in Lacaune sheep in heterozygotes [32]. In that study a single-strand conformation analysis of *Fecundity* loci in sheep revealed the presence of an FecX mutation on the X chromosome, corresponding to a G to A transition leading to the substitution of cysteine 53 to tyrosine in the mature domain of the *BMP15* gene. Heterozygous carriers showed increased ovulation rates and multiple births, but homozygous ewes exhibited POI with “streaked” ovaries, phenocopying our present clinical observation in humans. The follicle histology in C53Y homozygote sheep reveals enlarged oocytes and a disorganized granulosa layer, compatible with aberrant communication between the oocyte and the surrounding GCs. However, the ovaries are not devoid of follicles. In contrast to the sheep model, in which twinning or hyperfecundity were associated with heterozygosity for this mutation, we found no evidence of higher fertility rates or hyperfecundity in the studied family. The presence of follicles in the sheep model may have human clinical implications as early ovarian follicles (that may be preserved) may exist in prepubertal ovaries of humans that harbor the C320Y mutation, despite the relatively low AMH levels found here in the prepubertal girls homozygous for the mutation.

Of note is the use of the COV434 GC line and not oocytes that naturally secrete BMP15 to evaluate the mutant *BMP15* function in this study. Given the ethical difficulties in obtaining and using human oocytes, we have opted for COV434 GC lines as they are the natural target of BMP15 protein and express the receptors and necessary SMAD1/5/8 cascade essential for transduction of the BMP15 signal. A similar protocol for assessment of mutated *BMP15* has been used in previous studies [13, 20, 33, 34].

In summary, this work provides functional proof for the role of BMP15 in human folliculogenesis. The clinical phenotype of primary amenorrhea with reduced ovarian reserve reflected by reduced AMH levels and hypotrophic ovaries is compatible with the known effects of *BMP15* on GC proliferation and an arrest in follicle maturation. However, whether the effect of the studied mutation is due to aberrant formation or synergy with GDF9 remains to be elucidated.

Clinically, these results may allow pregestational prenatal and earlier diagnosis, which may indicate ovum preservation. The ovine phenocopy poses an exciting and unparalleled opportunity for further functional studies of a human disease in an existing animal model.

### Conclusion

In this study a homozygous *BMP15* mutation is ascertained as a genetic etiology for OD. The novel C320Y homozygous mutation in human *BMP15* is shown to cause primary amenorrhea and OD in human due to aberrant BMP15 signal transductions in GC, which is phenocopied by the ovine animal model. Further studies to determine whether these mutations exert their effect through abrogation of the Smad1/5 pathway or by inhibiting the formation or action of *Cumulin* and abrogation of the Smad2/3 pathway are underway.

## Data Availability

Original data generated and analyzed during this study are included in this published article or in the data repositories listed in “References.”

## Abbreviations

3D: 3-dimensional
AMH: antimüllerian hormone
ANOVA: analysis of variance
BMP15: bone morphogenic protein 15
BRE: BMP-responsive element
GC: granulosa cell
OD: ovarian dysgenesis
POI: primary ovarian insufficiency
WES: whole-exome sequencing
WT: wild-type
